# Illness meanings and experiences for pre-ulcer and ulcer conditions of Buruli ulcer in the Ga-West and Ga-South Municipalities of Ghana

**DOI:** 10.1186/1471-2458-12-264

**Published:** 2012-05-11

**Authors:** Mercy M Ackumey, Margaret Gyapong, Matilda Pappoe, Cynthia Kwakye-Maclean, Mitchell G Weiss

**Affiliations:** 1 School of Public Health, College of Health Sciences, University of Ghana, Legon, Ghana; 2 Swiss Tropical and Public Health Institute, Basel, Switzerland; 3 University of Basel, Basel, Switzerland; 4 Dodowa Health Research Centre, Ghana Health Service, Ghana; 5 Ga-West Municipal Health Administration, Amasaman, Ghana

## Abstract

**Background:**

Ghana is a Buruli ulcer (BU) endemic country yet there is paucity of socio-cultural research on BU. Examining distinctive experiences and meanings for pre-ulcers and ulcers of BU may clarify the disease burden, illness experience and local perceptions of causes and spread, and environmental features of BU, which are useful to guide public health programmes and future research. This study aimed to explain local meanings and experiences of BU for persons with pre-ulcers and ulcers in the Ga-West and Ga-South municipalities in Accra.

**Methods:**

Semi-structured interviews based on the Explanatory Model Interview Catalogue framework were administered to 181 respondents comprising 15 respondents with pre-ulcers and 166 respondents with ulcers. The Wilcoxon rank-sum test was used to compare categories of illness experiences (PD) and perceived causes (PC) among respondents with pre-ulcer and ulcer conditions. The Fisher’s exact test was used to compare the most troubling PD and the most important PC variables. Qualitative phenomenological analysis of respondents’ narratives clarified illness experiences and meanings with reference to PC and PD variables.

**Results:**

Families of respondents with pre-ulcers and the respondents themselves were often anxious about disease progression, while families of respondents with ulcers, who had to give care, worried about income loss and disruption of school attendance. Respondents with pre-ulcers frequently reported swimming in ponds and rivers as a perceived cause and considered it as the most important PC (53.3%). Respondents with ulcers frequently attributed their BU illness to witchcraft (64.5%) and respondents who claimed they had no water contact, questioned the credibility of health messages

**Conclusions:**

Affected persons with pre-ulcers are likely to delay treatment because of social and financial constraints and the absence of pain. Scepticism on the role of water in disease contagion and prolonged healing is perceived to make ideas of witchcraft as a PC more credible, among respondents with ulcers. Health messages should address issues of locally perceived risk and vulnerability. Guided by study findings, further research on the role of environmental, socio-cultural and genetic factors in BU contagion, is also needed to clarify and formulate health messages and strengthen public health initiatives.

## Background

Buruli ulcer (BU) caused by the environmental pathogen *Mycobacterium ulcerans,* is a debilitating skin disease [1-3]. However, the mode of BU transmission remains unclear [4]. Socio-cultural studies of malaria [5], tuberculosis [6] and lymphatic filariasis [7] show how socio-cultural factors influence illness perceptions, experiences and outcomes. An assessment of illness experiences of BU is needed to clarify illness-related problems and concerns, and the distinctive, psychological, social and socio-economic impact of pre-ulcer and ulcer conditions. These assessments are useful to reveal the social and economic burden of BU, local needs and information gaps, and to guide pragmatic public health interventions for treatment, that take into consideration the social, cultural and environmental contexts of affected persons.

Since the discovery of BU in the 1900s [8], there have been several epidemiological studies [2,9-13]. Some studies have also highlighted water contact as a risk factor for BU illness which form the basis for health education messages that emphasise water contagion from unpotable sources as a risk factor for BU infection [2,14-18]. Yet, there is a paucity of socio-cultural research on BU, particularly in Ghana where the disease was first reported in 1971 [19]. The extent of the BU-related disease burden for pre-ulcer and ulcer conditions cannot be explained adequately by epidemiological studies alone. Few studies have indicated the impact of the socio-economic burden of the BU illness on productivity, family welfare, education and treatment [20-23] and have indicated the influence of perceived spiritual causes on help-seeking behaviour [22,24,25]. Moreover, there is little research on the implications of the BU disease burden on gender roles, gender dimensions of care and implications for productivity, and family welfare.

Health programmes often assume that BU public health initiatives based on scientific research are well understood by the affected community but this is not necessarily so. Socio-cultural studies of pre-ulcer and ulcer conditions of BU are therefore indispensable to clarify issues of susceptibility to infection, knowledge gaps and the impact of BU on the individual as well as the family. These assessments are critical for designing effective BU control programmes that are sensitive to the cultural and environmental context of endemic communities. The purpose of this study was to explain local meanings and experiences of BU infection for respondents with pre-ulcer and ulcer conditions in the Ga-West and Ga-South municipalities in the Greater-Accra region of Ghana.

## Methods

### The study area

This study was conducted from November 2008 to July 2009 in the Ga-West and Ga-South Municipalities of the Greater Accra region. The 2009 population estimates for the Ga-West municipality (GWM) was 215,824 (Ga-West Municipal Health Directorate, annual report, unpublished). About 60% of the population reside in 200 rural scattered communities; the rest of the land area is peri-urban and densely populated. The GWM shares boundaries with the Ga-South municipality (GSM) to the west, and has an estimated population of 210,727 located in about 362 communities, mainly peri-urban [26]. Both municipalities have a similar population structure; 35% of the population are below the ages of 15 years and 65% are 15 years-of-age and above. The major BU medical treatment centres are the Amasaman Hospital (AH) and the Kojo Ashong Clinic (KAC) in the GWM, and the Obom Health Centre (OHC) in the GSM. The AH is the main referral centre for BU treatment in these municipalities. These municipalities are the fifth most endemic with respect to BU, yet have the highest case-loads in terms of healed and active lesions [9]. BU continues to be a major cause of morbidity in these two municipalities with increasing numbers of related disabilities.

### The study sample and sampling strategy

To identify as many BU affected persons as possible, a sample of 181 respondents was obtained from 67 communities and 3 BU treatment centres. Respondents who had pre-ulcer conditions rather than ulcers, at the time of the study were classified as ‘pre-ulcers’. AH admits approximately 90 persons with BU infection each year. Based on these estimates, we enlisted all BU patients receiving treatment at the AH, KAC and OHC and all affected persons from 67 endemic communities. The intention to interview respondents from health facilities and communities enabled us to obtain an adequate sample of BU-affected people to compare pre-ulcer and ulcer conditions.

A list of endemic communities was obtained from the municipal health directorates of the GWM and GSM. These communities were visited and community participants were located with the assistance of community-based surveillance volunteers (CBSVs) who kept registers of all affected persons. Neighbouring communities (which were not listed as endemic), were entered and affected persons were located and interviewed with the help of CBSVs. The research team compiled a register of persons that had been interviewed to avoid duplicate interviews of the same respondent. Community participants who indicated that they were out-patients were checked on our register to ascertain if they had already been interviewed in the health centres. Schools in sampled communities were also visited, and with the permission of the head teacher and class teacher, a WHO BU picture guide [27] was shown to the children. Children who admitted to having suspicious lesions were screened by health personnel from the KAC for confirmation. In endemic areas with a long history of BU infection, trained health workers are capable of identifying cases using the WHO classification [27]. With the exception of children less than 5 years-of-age whose parents acted as proxy respondents, older children were interviewed first and subsequently care-takers, who were invariably parents of children. Coded responses reflected consensus opinion.

### The explanatory model interview catalogue interview

A semi-structured interview schedule was developed to study and clarify socio-cultural concepts of illness from the perspective of persons who are directly affected [28]. This explanatory model interview schedule was based on the framework of the Explanatory Model Interview Catalogue (EMIC) for cultural epidemiology. Like other EMIC interviews, this one had a common core structure to examine illness experiences and meanings of BU.

The design of the EMIC instrument was informed by preliminary ethnographic field experience, focus group discussions and earlier studies [22,23]. The instrument was developed in English and translated for interviews in the local Ghanaian languages, spoken by respondents in the study areas, (Ga, Ewe and Twi). The EMIC interviews elicited responses for illness meanings and experiences for BU. Questions on illness meanings (PC) explored various ideas about causes for BU such as ingestion, injury, environmental, behavioural and spiritual. Information on illness experiences (PD) was obtained by asking questions about physical conditions, social, psychological or emotional problems and the impact on caretakers work or school, to provide support. Children were not asked PD questions that were irrelevant, such as questions related to marriage, income and employment. To enable a comparative analysis of PD and PC variables for pre-ulcers and ulcers, the same EMIC interview was administered to all respondents. Respondents’ narratives to open-ended questions elaborated and explained responses to coded categories.

### Data Management and analysis

Categorical and numeric data from the EMIC interviews were double entered using EPI Info (Centers for Disease Control and Prevention, Atlanta, GA, USA, version 3.4.1) and subsequently cleaned and analysed using STATA 10.1 data analysis and statistical software (StataCorp, Lakeway Drive, College Station, Texas). The analysis compared illness experiences and meanings for respondents with pre-ulcers and ulcers to elucidate similarities or differences in the ways that respondents experienced and explained their conditions.

Total frequencies and prominence of variables for PD and PC were compared for pre-ulcers and ulcers. Responses were classified on a prominence scale as follows: a spontaneous response was assigned a value of 2, a response after a probe (in the absence of a spontaneous answer) a value of 1, and no response, a value of 0. Respondents were asked to indicate the most troubling PD and the most important PC. These responses contributed an additional value of 3. A cumulative prominence (ranging from 0–5) was then computed for PD and PC variables which facilitated a comparative analysis for pre-ulcers and ulcers. The Wilcoxon rank-sum test for non-parametric data was used to compare the ranked prominence of PD and PC variables for pre-ulcers and ulcers. The Fisher’s exact test was also used to compare the frequency of each reported category of most troubling PD and most important PC. Individual cultural epidemiological variables for PD and PC were also grouped thematically for analysis and comparison of overarching concepts (physical conditions, social problems and psychological problems for PD; ingestion, illness/injury, environmental, behaviour and spiritual for PC). Furthermore, we examined the perceived seriousness of BU, the social effect of respondents’ illness conditions on the family and the gender dimensions of care for pre-ulcer and ulcer conditions.

Narrative data were transcribed into English during the interview by the data collector and entered into Microsoft Word 2002. These narrative data were analysed with MAXQDA 10 (Verbi Software Consult Sozialforschung, GmbH, Marburg, Germany) software for textual analysis. Phenomenological analyses of PD and PC were compared for selected respondents’ narratives based on thematic deductive coding. Narratives were selected for qualitative analysis according to coded responses imported into the qualitative data programme (MAXQDA). This approach clarified essential features of explanatory variables associated with illness meanings and experiences for pre-ulcers and ulcers.

### Ethical considerations

Verbal informed consent was obtained from all adult respondents and parental caretakers or guardians of children. The study was approved by the ethical review committee of the Ministry of Health, Ghana, and the ethics commission of Basel, Ethikkommission beider Basel (EKBB), in Switzerland.

## Results

### Sample characteristics

A total of 181 respondents were interviewed. The majority of respondents had ulcers (91.7%) and only 8.3% had pre-ulcers. Respondents with pre-ulcers and ulcers had similar background characteristics. Most respondents had at least completed primary school. Very few respondents were skilled or professional workers and very few had regular income (Table 1).

**Table 1:** Demographic characteristics of respondents

**Demographic Characteristics**	**Pre-ulcer N = 15**	**Ulcer N = 166**	**Total N = 181**
	**N (%)**	**N (%)**	**N (%)**
**Sex**			
Males	7 (46.7)	80 (48.2)	87 (48.1)
Females	8 (53.3)	86 (51.2)	94 (51.9)
**Age of respondents**			
Minimum age	6	3	3
Maximum age	64	87	87
Mean age	19	22.8	22.46
Standard deviation	14.9	18.3	18.07
**Education**			
No education	3 (20.0)	39 (23.5)	42 (23.2)
Primary	8 (53.3)	90 (54.2)	98 (54.1)
Secondary and above	4 (26.7)	37 (22.3)	41 (22.7)
**Occupation**			
Pupil/student	11 (73.3)	89 (53.6)	100 (55.2)
Unskilled labourer	3 (20.0)	44 (26.5)	47 (26.0)
Skilled labourer/Professional	1 (6.7)	12 (7.2)	13 (7.2)
Unemployed	0	14 (8.4)	14 (7.7)
Other (too young to be either employed or in school)	0	7 (4.2)	7 (3.9)
**Income**			
Regular and dependable	5 (33.3)	29 (17.3)	34 (18.8)
Uncertain/ Cannot tell	4 (26.7	65 (39.2)	78 (43.1)
Irregular	6 (40.0)	72 (43.4)	69 (38.1)
**Marital status**			
Never married	11 (73.3)	114 (68.7)	125 (69.0)
Married	4 (26.7)	38 (22.9)	42 (23.2)
Separated / divorced	0	5 (3.0)	5 (2.8)
Widowed	0	9 (5.4)	9 (5.0)

### Burden of BU and impact on family well-being

Features of the impact of BU were disrupted livelihoods, loss of income, absence from work or school for care, and anxiety about disease progression. While respondents with pre-ulcers emphasised the point that their families were more concerned about the progression and course of their illness (66.7%), respondents with ulcers emphasised loss of income as the main concern of family members (80.1%) (Table 2).

**Table 2:** Impact of respondent’s illness condition on family

**Illness impact**				**Pre-ulcers, N = 15**					**Ulcers, N = 166**
	**Total %**	**Spon. %**	**Mean Prominence**	**Total %**	**Spon. %**	**Mean Prominence**	**P-values**
Loss of income	33.3	13.3	0.47	80.1	60.8	1.41	***
Sadness, anxiety and worry	60.0	6.7	0.67	71.7	28.9	1.01	
Concern about course of illness	66.7	26.7	0.93	81.9	35.5	1.17	
Miss work for care-taking	26.7	0.0	0.27	85.5	54.2	1.40	***
None	26.7	20.0	0.47	1.2	1.2	0.02	***
Left the family without support	6.7	6.7	0.13	1.8	1.8	0.04	

Categories reported by less than 5% of respondents are not included in the table. Columns indicate total reported responses in percentages, spontaneously reported responses in percentages and the mean prominence. Total reported values include combined spontaneous and probed responses. The mean prominence was calculated based on assigned values to each reported category (2 = spontaneous response, 1 = probed response, 0 = not reported). The Wilcoxon ranksum test was used to compare mean prominence for pre-ulcers and ulcers (*p ≤ 0.05, **p ≤ 0.01, ***p ≤ 0.001).

Respondents with nodules indicated in their narratives that their condition did not pose any threat to their well-being and family welfare since they were in no pain, could use affected limbs, and therefore were able to perform their daily routines of school and work, without any limitation. Family members of respondents with pre-ulcer conditions worried about the progression of the illness of their relatives. They were concerned about the outcome of *swollen* (oedematous) limbs or plaques and nodules that were likely to progress into ulcers with debilitating consequences of pain, disability and high costs of care. Narratives of respondents with ulcers referred to various effects of their condition on their family. These included disruption of work to provide care in the hospital and at home, and depletion of family income and resources for treatment costs. Family members of respondents with ulcers too were often concerned about disease progression and prolonged treatment, and the likelihood of disability.

### Gender dimensions of BU-burden and care

Socially constructed gender roles of care and work affected livelihoods, income and education of those providing care for sick relatives. The socio-economic status of families also worsened if the affected person was the main income-earner. Generally, for respondents with ulcers, mothers (52%) were more likely to miss work for caretaking than fathers (6%); daughters (7.2%) were more likely to stay away from school than sons (0.6%), and more sisters (8.4%) than brothers (1.2%) stayed at home to care for sick relatives. Similarly, pre-ulcer respondents with plaques and oedematous lesions also identified mothers as care-givers. The following illness narrative explains how the loss of livelihoods, anxiety, and the need for care affects the social and economic well-being of the family.

"It started as a hard boil (nodule). I showed it to a health worker at Hobor (a community in the GSM). He told me it was Buruli ulcer and said I should go to the hospital. I did not have enough money then, so I stayed at home for 3 weeks before going to the hospital. I am the bread winner of the family and now I am in hospital. My daughter comes here occasionally with food and money for me, and to wash my bandages. Since I am not working, my parents send me money and some provisions occasionally. When my parents do not have money, they do not send anything."

"(28-year-old female respondent)"

### Patterns of distress

Respondents with pre-ulcers and ulcers expressed their distress differently (Table 3). Those with pre-ulcers frequently reported psychological or emotional problems (86.7%), particularly anxiety (66.7%) and physical problems, mostly pain (66.7%). Psychological or emotional problems were mentioned as the most troubling category of distress and were more prominent for pre-ulcers. Pain was often associated with oedematous lesions. For ulcers, physical problems (98.2%) were frequently and more prominently reported. Pain and problems with mobility or use of affected limbs were physical problems that respondents with ulcers emphasised as distressing (Table 3). Disrupted education was the most frequently reported social problem.

**Table 3:** Reported categories of distress for respondents with pre-ulcers and ulcers

**Categories of distress**					**Pre-ulcers, N = 15**						**Ulcers, N = 166**
	**Total %**	**Spon. %**	**Most important %**	**Mean Prominence**	**Total %**	**Spon. %**	**Most important %**	**Mean Prominence**	**P-values**
**Physical problems**	**80.0**	**60.0**	**13.3**	**1.80**	**98.2**	**94.6**	**46.4**	**3.32**	***
Fever	26.7	13.3	0.0	0.40	28.9	9.6	0.6	0.40	
Pain	66.7	46.7	6.7	1.33	86.8	72.3	14.5	2.02	*
Smell	6.7	0.0	0.0	0.07	66.3	14.5	1.2	0.84	***
Weight loss	13.3	0.0	0.0	0.13	56.6	18.7	0.6	0.77	***
Loss of appetite	20.0	0.0	0.0	0.20	43.4	9.0	0.0	0.52	
Weakness	26.7	6.7	0.0	0.33	37.4	8.4	0.0	0.46	
Problems with mobility and use of affected limbs	40.0	13.3	6.7	0.73	83.1	68.7	29.5	2.40	***
Condition is ugly	13.3	6.7	0.0	0.20	41.6	3.6	0.0	0.45	*
**Social problems**	**40.0**	**26.7**	**40.0**	**1.87**	**89.2**	**67.5**	**36.1**	**2.65**	*
Rejection from family	0.0	0.0	0.0	0.00	19.3	2.4	0.0	0.22	
Rejection by friends / peers	6.7	0.0	0.0	0.07	22.9	3.6	0.0	0.27	
Disrupted education	33.3	20.0	33.3	1.53	56.6	45.2	26.5	1.81	
Loss of income	6.7	6.7	6.7	0.33	38.0	22.3	9.6	0.89	*
**Psychological /emotional**	**86.7**	**20.0**	**46.7**	**2.47**	**78.9**	**28.9**	**16.3**	**1.57**	
Anxiety	66.7	13.3	33.3	1.80	61.5	15.1	9.6	1.05	
Fear of surgery	20.0	0.0	0.0	0.20	18.7	3.6	1.8	0.28	
Embarrassed about condition	20.0	6.7	13.3	0.67	49.4	12.7	4.2	0.75	
**Miscellaneous**	**0.0**	**0.0**	**0.0**	**0.00**	**12.7**	**12.7**	**1.2**	**0.29**	
Disrupted life and sleeplessness	0.0	0.0	0.0	0.00	12.7	12.7	1.2	0.29	

Categories reported by less than 5% of respondents are not included in the table. Grouped categories (in bold) computed from responses. Columns indicate total reported responses in percentages, spontaneously reported responses in percentages and the mean prominence. Total reported values include combined spontaneous and probed responses. The mean prominence was based on assigned values to each reported category (3 = Most troubling distress, 2 = spontaneous response, 1 = probed response, 0 = not reported). The Wilcoxon ranksum test was used to compare means for pre-ulcers and ulcers (*p ≤ 0.05, **p ≤ 0.01 ***, p ≤ 0.001).

Narratives showed that respondents with pre-ulcers were often anxious about the progression of their illness to ulcers. This concern was influenced by prior knowledge of the debilitating nature of illness progression from pre-ulcers to ulcers, uncertainty of disease outcomes and concern about transportation costs for treatment. One respondent worried that *it* (nodule) *will become a sore just like those of other people who already have it, and my leg will be cut.* Respondents who were not familiar with pre-ulcer conditions too were often anxious about the outcome of their illnesses. Desperation and desire for clarification of their conditions and relief led them to seek advice and help from friends and family, and to shop for care from various providers, such as herbalists, church, and private health practitioners. Many respondents with pre-ulcers did not want normal work and school attendance to be disrupted. Therefore, they used itinerant providers who could provide services in the respondents’ homes.

Like respondents with pre-ulcers, respondents with ulcers who were in school often worried about their education being disrupted (56.6%) and expressed anxiety (61.5%) about the outcome of their ulcers. Narrative accounts of respondents with ulcers revealed that their distress was influenced by a combination of physical, social and psychological problems. For example, anxiety was often triggered by the intensity of pain and the inability to use affected limbs or move around easily, which hampered work and school. As recourse for cure, and to continue with work and school, respondents with ulcers too, preferred help from itinerant providers such as herbalists, private health practitioners and other government employed health workers, who provided care in their homes after work. As their illness conditions worsened and pain intensified, respondents were compelled to seek help from the municipal health facilities. A female respondent explained how pain and immobility had affected her livelihood and income. Desperate to recover quickly and to continue working and taking care of her children, she used various providers and eventually used medical care.

"I have been suffering for some time now. My leg hurts and I cannot walk properly with this leg. I used to be an okra farmer, but I cannot farm anymore. I do not make money anymore to take good care of my children. I bought all kinds of drugs from the people who sell medicine (drug peddlers), but they did not work. I visited so many places for help. I bought any medicine I heard of, but none of them helped me. My pastor told me to go to the health centre as it was getting worse but I rather went to see a herbalist, but his treatment did not work, the sore was getting bigger and bigger. I wanted to get well quickly to go back to farming. I finally came to the hospital."

"(28-year-old female respondents)"

Likewise, a mother’s anxiety about her son’s condition focused on the fear that he might drop out of school. Aside from her child’s distress, she also bemoaned her absence from the home because of care, loss of work and the gradual dwindling of her trading capital, and eventual poverty.

"Now that he has Buruli ulcer when will he recover in order to go back to school? Sometimes I am afraid that this is it; he may never go back to school. Since I am his mother, I have to be with him at the hospital. I had to spend Christmas here in the hospital, away from the family. I have stopped trading and my capital which was a loan from the bank has been spent on looking after my son. Meanwhile, it is still building-up interest. How am I going to pay back the money when I have stopped work?"

"(Mother of 9-year-old male child)"

### Perceived causes

Respondents mentioned a variety of causes to explain their illness. For both pre-ulcers and ulcers, perceptions of causes were based on observation, behaviour, the influence of health messages on contagion, and the logic of explanations they had for their illness.

Respondents with pre-ulcers frequently and prominently reported behaviour-related causes, particularly swimming in ponds and rivers which was also considered as the most important perceived cause (53.3%) (Table 4). Respondents, who reported swimming in ponds and rivers, linked their condition to their own risky behaviour. Some respondents, who said they had no contact with water bodies, questioned the credibility of health messages that linked contagion to contact with unclean water. They referred to the absence of the disease in other community members with whom they shared the same water sources (rivers and ponds). Furthermore, about half of respondents with pre-ulcers attributed their illness to drinking unclean water (53.3%), and about a third (33.3 %) of respondents could not tell the cause of their illness.

**Table 4:** Reported categories of perceived causes for respondents with pre-ulcers and ulcers

**Perceived causes**					**Pre-ulcers, N = 15**						**Ulcers, N = 166**
	**Total %**	**Spon. %**	**Most important %**	**Mean Prominence**	**Total %**	**Spon.%**	**Most important %**	**Mean Prominence**	**P-values**
**Ingestion**	**53.3**	**26.7**	**6.7**	**1.00**	**33.7**	**11.5**	**4.2**	**0.58**	
Drinking unclean water	53.3	26.7	6.7	1.00	33.7	11.5	4.2	0.58	
**Illness/ Injury**	**20.0**	**0.0**	**0.0**	**0.20**	**44.0**	**27.1**	**9.0**	**0.98**	*
Prone to illness	13.3	0.0	0.0	0.13	13.9	4.8	0.6	0.20	
Insect bites	13.3	0.0	0.0	0.13	7.2	2.4	0.6	0.11	
Scratches on skin	13.3	0.0	0.0	0.13	14.5	7.2	1.8	0.27	
Weakness of blood	20.0	0.0	0.0	0.20	38.6	24.1	6.0	0.81	
**Environmental**	**40.0**	**6.7**	**0.0**	**0.47**	**34.3**	**15.7**	**4.2**	**0.63**	
Poor sanitation	40.0	0.0	0.0	0.40	27.7	10.2	1.2	0.42	
Poor personal hygiene	26.7	0.0	0.0	0.27	21.1	5.4	2.4	0.34	
Exposure to sand	20.0	6.7	0.0	0.27	23.5	7.8	0.6	0.33	
**Behaviour**	**60.0**	**40.0**	**53.3**	**2.60**	**48.2**	**28.9**	**18.1**	**1.31**	
Swimming in ponds and rivers	60.0	40.0	53.3	2.60	43.4	25.9	18.1	1.23	*
Contact with animals	6.7	6.7	0.0	0.13	9.0	3.0	0.0	0.12	
**Spiritual**	**20.0**	**6.7**	**0.0**	**0.27**	**64.5**	**51.2**	**39.7**	**2.35**	**
Witchcraft	20.0	6.7	0.0	0.27	64.5	51.2	39.7	2.35	**
**Miscellaneous**	**46.7**	**46.7**	**40.0**	**2.13**	**31.9**	**31.9**	**24.7**	**1.38**	
Cuts, abrasions, unexplained swelling of limbs	13.3	13.3	13.3	0.67	8.4	8.4	6.6	0.37	
Cannot say/ uncertain	33.3	33.3	26.7	1.47	24.1	24.1	18.1	1.02	

Categories reported by less than 5% of respondents were not included in the table. Grouped categories (in bold) computed from responses. Columns indicate total reported responses in percentages, spontaneously reported responses in percentages and the mean prominence. Total reported values include combined spontaneous and probed responses. The mean prominence was calculated based on assigned values to each reported category (3 = Most important perceived cause, 2 = spontaneous response, 1 = probed response, 0 = not reported). The Wilcoxon ranksum test was used to compare mean prominence for pre-ulcers and ulcers (*p ≤ 0.05, **p ≤ 0.01, ***p ≤ 0.001).

Some respondents with pre-ulcers, who remembered how their illness started, were certain that they had no water contact. They attributed their illness to various factors like scratches, stings, abrasions and unexplained swellings of the limbs. Those respondents, who related their condition to bad drinking water, based this idea on health information from health workers, community-based surveillance volunteers and teachers. They admitted however, to drinking unclean water from rivers, ponds and dug-out wells and explained that they had no other option.

Respondents with ulcers emphasised witchcraft as a likely perceived cause and the most important perceived cause for their illness. Aside from such spiritual causes, swimming in ponds and rivers (43.4%), weakness of blood (38.6%) and drinking unclean water (33.7%) were also mentioned (Table 4). Like respondents with pre-ulcers, some respondents with ulcers could not tell the cause of their condition (24.1%).

Respondents’ narratives related ideas of witchcraft to a variety of other factors. These included: The absence of a logical explanation for infection within the context of health messages that emphasised contact with aquatic sources as a risk factor for contagion (especially when other persons exposed to risk factors like swimming, fishing and bathing in rivers were never infected); inability to explain the cause of the disease; progression of abrasions, small cuts and swellings into debilitating ulcers that took a long time to heal. Some parents could not understand how children, who were too young to swim and therefore had no contact with aquatic sources, were infected. An adult respondent explained her choice for medical care after a recurring BU infection. She mentioned witchcraft as a perceived cause of her illness and dismissed water contact as a plausible explanation:

"When it happened the first time, I tried herbal treatment and I really suffered before I got cured. So when it happened this time, I decided to go to the hospital. We have a pond in this village and no one swims or wades in this pond. I am a neat person and my house and compound are always clean. I do not swim or wash in bad water. So I don’t believe that this disease is from the water as the nurses here are saying. I believe that this condition is due to witchcraft because that is what witches do; they destroy people’s lives. This disease is terrible, it cripples you and ties you down for months and even years. We will be happy if a stronger and faster treatment can be found for this illness."

"(45-year-old female respondent)"

Narratives indicated that scepticism of health messages, and reference to witchcraft as a PC, did not prevent respondents from using medical care. Illness experiences (PD), persistence of the lesion and failure to recover, and awareness of medical care for BU, influenced their choice of medical care. However, witchcraft-related explanations prejudiced notions of transmission and prevention. Many respondents with pre-ulcers (40.0%) and ulcers (50.0%) stated that their conditions could not be prevented because *witchcraft cannot be stopped.* Nevertheless, respondents with pre-ulcers (56.7 %) and ulcers (46.4%) mentioned *avoiding swimming and bathing in rivers and ponds* as an effective preventive measure. Narratives revealed that this information was obtained from health messages in the communities, school and health centres.

Like respondents with pre-ulcers, respondents with ulcers who mentioned water contact through swimming as a likely cause of their condition, blamed their associated behaviour for their illness and not a matter of lack of awareness. They explained that unclean water sources could not be avoided since there was no better alternative. These sources of water were used for bathing, washing, cooking and irrigation. Sometimes, during the rainy season, respondents had to wade through ponds as a thoroughfare.

Additionally, vulnerability to BU infection due to low immunity, referred to locally as *weakness of blood,* was mentioned as a possible cause of infection. Infected children, particularly those who had recurring lesions, were often described as having *weak blood.* A child explained why *weakness of blood* was more likely cause than water contact:

"I believe my condition is due to the weakness of my blood because all of us at home go to the river to fetch water and we use the same water. Why am I the only one to get infected? I had it some time ago and it has reoccurred."

"(16-year-old male respondent)"

## Discussions

To the best of our knowledge, this is the first study to compare illness meanings and experiences of BU for pre-ulcers and ulcers. Our study findings draw attention to the gendered burden of care for BU-affected persons and its impact on family welfare, work and school, the extent and nature of anxiety for pre-ulcers (on anticipated disease outcomes) and ulcers and disability from ulcers. Perceived causes for pre-ulcers and ulcers indicated the mismatch between professional and local ideas on disease contagion and revealed information gaps that need guidance from further research. Such scepticism about health messages, however, did not deter respondents from seeking medical care, which was influenced largely by illness experiences and the desire for recovery from persisting lesions. Study findings indicate a myriad of social, cultural, physical and behavioural issues associated with illness meanings and experiences. These findings highlight the need for health professionals to clarify messages on contagion and dispel fears of BU being perceived as a mysterious disease to encourage early medical treatment. Improving BU surveillance, case-detection and access to treatment and support groups and counseling services are important and could reduce the social and economic impact of BU.

### Study limitations and implications

Respondents were queried about illness experiences and meanings of their current conditions, and there were few respondents with pre-ulcers (15) compared with ulcers (166). Efforts to identify more respondents with pre-ulcers suggested that the low numbers of respondents with pre-ulcers may result from hastened progression to ulcers from cutting nodules and piercing oedematous tissue. This practice rapidly transforms pre-ulcer conditions into ulcers (Table 5). A recent study in a BU-endemic area in Ghana also showed fewer pre-ulcer cases (23.3%) than ulcer cases (76.7%) during an initial health-screening exercise. However, the situation reversed after one year of intensive health education [29]. Nevertheless, our findings are clearly relevant for our study communities and for other BU-endemic areas in Ghana.

**Table 5:** Local practices that transform pre-ulcers into ulcers

**Background characteristics of respondent**	**Narrative**	**Procedure adopted**
Father of 12-year-old female child	A herbalist cut the boil open and placed a herbal dressing on it.	Nodule was cut
Father of 4-year-old male child	We took him (son) to see his grandfather who cut the boil and it became a sore. His grandfather has been cutting other people’s boils that is why we took him to see him.	Nodule was cut
Father of 4- year-old male child	The boil became big and we cut it open and placed some herbal preparations on it.	Nodule was cut
A 13-year-old male respondent	My uncle used a sharp object to cut the boil so that the blood could come out. Then he applied some black powder and put some in alcohol for me to drink.	Nodule was cut
A 26-year-old female respondent	I burst the boil because I did not know what it was and then I cleaned the sore everyday with hot water.	Nodule was cut
A 48-year-old adult male respondent	My father put some herbs on the boil to open it up.	Herbs applied to the boil to open it up
An 18-year-old female respondent	My grandmother ground herbs mixed with salt and placed it on the boil so that it could burst.	Herbs applied to the boil to open it up
A 43-year-old female respondent	I had a swelling on my ankle for one week. My husband slit it and then it gradually became a big sore.	Oedema cut open

*Narrative data of other respondents with ulcers (not presented in this table) suggests that herbal preparations were placed on nodules, oedemas and plaques to open up the skin to expose the necrotic tissues. Subsequently herbal dressings were applied to the exposed tissues.

### Gender roles, gender dimensions of care and impact on family welfare

Because pre-ulcer conditions are normally painless and less debilitating than ulcers, medical care is often delayed. However, owing to the incapacitating nature of ulcers and prolonged healing required care for affected persons is considerable [30,31]. The gendered nature of care in our study has far reaching social and economic implications for the care-givers. First, when mothers and spouses are pre-occupied with caring for sick relatives, they have less capacity for other productive work which may jeopardise the welfare of the family, particularly young children.[21,30]. The absence of children from school because of their own illness or a need to care for others has serious implications for their future development and economic empowerment [32]. Economic constraints require affected persons who were the main income-earners to choose between medical and herbal treatment, and alternatives, considering the economic well-being of the family.

Health care providers should ensure that community members understand the benefits of early treatment to minimise suffering and the need for extended care. As much as possible, over-reliance on family members to provide care in the health facilities should be discouraged. Perhaps, young people from the national youth employment programme [33], employed as health extension workers, could assist with the care of young patients and other patients with disability, providing community-based social support that relieves the burden on the family.

### Substantial psychological and social impact of BU among respondents

The frequency with which anxiety about disease progression was reported by respondents with pre-ulcers and ulcers suggests high levels of awareness about BU, the debilitating consequences if pre-ulcers are not treated early, and the implications of the cost of treatment. Medical care is free in our study communities [23,30]. It is therefore expected that illness experiences associated with pre-ulcers and ulcers would prompt early medical care. However, some respondents delayed treatment for pre-ulcers as long as there was no pain or disability. Respondents with ulcers also delayed medical treatment irrespective of their pain, anxiety and disability until they could acquire enough money for transport and food while hospitalised.

BU is known to affect impoverished rural communities with poor access to health facilities, thus exacerbating poverty and suffering [2,3,34], and limiting opportunities for education and productivity [20,21]. The social and economic impact of the BU illness is critical because the majority of the people in our study communities depend on subsistence agriculture [30]. Research shows that in the Ga-West municipality a patient with a nodule may be hospitalised for 74 days, and a patient with an ulcer may spend nearly a year (301 days), on average, in treatment. This same study [30] also revealed that families and BU-affected persons sold assets and properties like farm equipment and livestock, used up savings and borrowed money to pay for transport and food while in treatment at the hospital, and for the upkeep of the family. Relatives of respondents and respondents, who had to miss work or school understandably bemoaned the socio-economic impact of BU affecting their own well-being and the welfare of the family.

### Support groups and counselling services

Peer support groups for affected persons, mothers and care-givers may represent a cost-effective and culturally appropriate intervention for the psychological, social and medical management of BU, particularly in geographically dispersed communities such as our study area. The benefits of support groups or networks are far reaching and include information sharing on appropriate help-seeking behaviour, encouragement to initiate timely treatment and adherence to treatment [35-39]. Peer support groups of former or current affected persons may serve as points of psychological encouragement and counselling and are vital for sharing illness experiences and learning coping strategies, thus limiting the effects of stigma or social exclusion [36-41].

### Perceived causes and implications for BU prevention and the role of health education

The high proportion of respondents in our study that reported witchcraft as a perceived cause (20% of respondents with pre-ulcers and 64.5% of respondents with ulcers) is much higher than indicated by findings from an earlier study of BU knowledge in the Ga-West and Ga-South municipalities in which 5.2% of respondents mentioned witchcraft- related causes [22]. Explanations for witchcraft-related causes in our study were based on the sudden and inexplicable swelling of limbs, and the progression of pre-ulcers into painful ulcers which healed slowly and led to deformities. Perception of spiritual factors are likely when BU disease is prolonged [24,42,43]. Linking BU infection with a spiritual cause is likely to influence help-seeking from traditional healers to counteract the spell of the disease, especially spiritualists [13,25,43]. However, the majority of respondents in our study used herbalists and not spiritualists for treatment of their conditions, and not to liberate themselves from the spell of BU.

Furthermore, ideas of witchcraft as a perceived cause did not prevent our study respondents from seeking medical care. Local ideas about pervasiveness of witchcraft prejudiced them against some health messages. Scepticism about standard prevention strategies based on avoiding contact with rivers and ponds prevents a challenge to health professionals. Since slow healing of ulcers suggests ideas of witchcraft, health messages should emphasise that BU is an ordinary disease that will heal more quickly if treatment is initiated early during the pre-ulcer phase of infection.

### Early case-detection and disease surveillance for prompt medical care

For impoverished BU endemic areas, a comprehensive approach for prevention and treatment that addresses the health, social and economic impact of the BU illness would be ideal. Periodic screening in schools and communities by health professionals, CBSVs and teachers should endeavour to detect early cases for screening and treatment regularly [23,29]. Periodic screening might be useful in diagnosing all forms of skin trauma, lesions, stings and bites which may be unrecognised onset of BU and refer promptly for medical care. Teachers and community-based surveillance volunteers in our study communities have already been trained to screen school children and community members [23]. However, there is the need to strengthen this skill by re-training former CBSVs and teachers, and training new teachers that have been posted to these communities.

### Improving access to medical care

Health education, early screening and case detection alone may not achieve its goal of encouraging and sustaining early medical care and lessen the social and economic hardships, unless treatment centres are provided within reach of communities, ensuring easy access to treatment at minimum cost.

WHO-recommended antibiotics has been proven to shrink nodules and ulcers and prevent recurrences [23,44-46]. Surgery may also be required for oedematous lesions and plaques after antibiotic treatment. Decentralising health care by partnering with private health care providers to provide antibiotic treatment in close proximity to residences could minimise length of hospitalisation and socio-economic impacts. These strategies have been discussed in detail in previous papers [23,41]. Mobile health services may contribute to improving access to antibiotic treatment and should be integrated into the community-based health planning and services (CHPS) initiative. The CHPS concept is a national health policy initiative that aims to improve access to care and disease surveillance in poor, rural and dispersed communities. Health workers reside within a community and provide mobile health services and follow-up on patients within catchment areas [47]. Studies have documented the usefulness of such initiatives that combine screening, education and surveillance in disease control to minimise disease morbidity [48,49].

### Providing transport and feeding to encourage early treatment

Anxiety, experienced by respondents with pre-ulcers was linked to imminent progression of pre-ulcer conditions to ulcers, and the inherent costs of transports and feeding associated with treatment. BU public health programmes need to consider transport and feeding as a cost effective strategy to encourage early treatment seeking during the pre-ulcer phase of infection to minimise delayed treatment for ulcers that may require surgery and possibly prolonged healing and hospitalisation [50]. Since the host immune response is critical for BU disease progression and healing [1,4,51], feeding programmes may boost the immune system and improve treatment outcomes [50]. These feeding initiatives already exist in the Amasaman Hospital and need to be extended to other health facilities in the study area.

### Improving access to clean water

The absence of clean water for basic domestic and hygiene activities, such as washing, cleaning, cooking and bathing in our study communities explains why reliance on unclean infected sources persists. Most BU-endemic communities are rural and lack basic amenities, including clean water [52]. The continuous use of unclean water for domestic purposes, swimming and bathing, and small-scale fishing in rivers and ponds [53], defeats the purpose of health messages that emphasise contact with unclean water as a risk factor for BU infection. Providing clean water is likely to reduce BU infections considerably. Municipal authorities should solicit help from Non-Governmental Organisations (NGOs) to provide boreholes and other potable water systems.

### Needed research to explain the role of environmental factors for BU contagion

Recent environmental studies on BU transmission confirm the presence of *M. ulcerans* in aquatic environments [54,55]. Environmental factors have been mentioned as a cause of BU infection in previous socio-cultural studies [22,24], although it has been argued in one study that respondents’ views on the role of environmental factors in BU contagion were influenced by health messages rather than indigenous cultural ideas [24]. Although the mechanism of BU transmission remains unclear [3,4,56], health messages link disease contagion to water-related activities and encourage endemic communities to minimise water contact [2,18,21]. The empirical basis of these health messages is widely accepted and some respondents acknowledged their own risky behaviour as contributing to infection. Other respondents, however, were sceptical of these health messages. For them, messages failed to explain why persons with risky behaviour were not infected, and why others without such water contact nevertheless got BU. This shows a mismatch and an information gap between professional knowledge that requires credible bridging.

It has been argued that alternate explanations for BU transmission should be more widely acknowledged, especially direct skin contact with contaminated water [18,57-59] and the possible role of animal and anthropoid vectors [60,61]. Immunological research indicates that persons exposed to *M. ulcerans* might never develop the BU disease due to host immunity [4]. A deeper understanding of the social and environmental contexts of BU is needed, considering, for example, whether persons living in non-aquatic environments can be infected by insects [62]. Future environmental studies need to investigate transmission of *M. ulcerans* in non aquatic environments in endemic areas, to clarify health messages and appropriate community guidance. Clearer, credible explanations of transmission patterns will instill confidence in the health system, health professionals and health messages for effective public health action.

## Conclusions

The social burden of BU is enormous. Our study suggests that besides physical pain, disability and anxiety about the progression of the disease, BU affects livelihoods, interrupts education and jeopardises the welfare of affected families. Persons with pre-ulcer conditions are likely to delay treatment because of social and financial constraints and the absence of pain. Communities remain sceptical about the role of water in disease contagion, and these questions make ideas about witchcraft as a perceived cause more credible among people with ulcers. Study results suggest that health education messages should acknowledge locally perceived risk and vulnerability. Health education is not enough, however, and peer support groups are also needed to provide emotional and social support, to boost self esteem and to encourage early treatment. Since the mode of transmission remains unclear, further research on the role of environmental, socio-cultural and genetic factors in BU contagion is needed for practical and useful guidance for communities and to strengthen public health initiatives. Our study findings are relevant for other BU-endemic regions and communities in the country.

## Competing interests

The authors declare that they have no competing interests.

## Authors' contributions

MMA designed the study, collected and analysed field data and wrote the manuscript. MG and MP assisted in study design and editing of the manuscript. CKW assisted in the editing of the manuscript. MGW also designed the study, assisted with data analysis and editing of the manuscript. All authors read and approved the final manuscript.

## Pre-publication history

The pre-publication history for this paper can be accessed here:

http://www.biomedcentral.com/1471-2458/12/264/prepub

## References

[B1] Johnson PD Stinear T Small PL Pluschke G Merritt RW Portaels F Huygen K Hayman JA Asiedu K Buruli ulcer (M. ulcerans infection): new insights, new hope for disease control PLoS Med 2005 2 e108 15839744 10.1371/journal.pmed.0020108 PMC1087202

[B2] Marston BJ Diallo MO Horsburgh CR Diomande I Saki MZ Kanga JM Patrice G Lipman HB Ostroff SM Good RC Emergence of Buruli ulcer disease in the Daloa region of Cote d'Ivoire Am J Trop Med Hyg 1995 52 219 224 7694962 10.4269/ajtmh.1995.52.219

[B3] WHO Buruli ulcer: progress report, 2004–2008 Wkly Epidemiol Rec 2008 83 145 154 18437758

[B4] Portaels F Silva MT Meyers WM Buruli ulcer Clin Dermatol 2009 27 291 305 19362692 10.1016/j.clindermatol.2008.09.021

[B5] Ahorlu CK Koram KA Ahorlu C De SD Weiss MG Community concepts of malaria-related illness with and without convulsions in southern Ghana Malar J 2005 4 47 16188023 10.1186/1475-2875-4-47 PMC1262759

[B6] Weiss MG Somma D Karim F Abouihia A Auer C Kemp J Jawahar MS Cultural epidemiology of TB with reference to gender in Bangladesh, India and Malawi Int J Tuberc Lung Dis 2008 12 837 847 18544214

[B7] Gyapong M Gyapong JO Adjei S Vlassoff C Weiss M Filariasis in northern Ghana: some cultural beliefs and practices and their implications for disease control Soc Sci Med 1996 43 235 242 8844927 10.1016/0277-9536(95)00365-7

[B8] MacCallum P Tolhurst JC Buckle G Sissons HA A new mycobacterial infection in man J Pathol Bacteriol 1948 60 93 122 18876541

[B9] Amofah G Bonsu F Tetteh C Okrah J Asamoa K Asiedu K Addy J Buruli ulcer in Ghana: results of a national case search Emerg Infect Dis 2002 8 167 170 11897068 10.3201/eid0802.010119 PMC2732443

[B10] van der Werf TS van der Graaf WT Tappero JW Asiedu K Mycobacterium ulcerans infection Lancet 1999 354 1013 1018 10501380 10.1016/S0140-6736(99)01156-3

[B11] van der Werf TS van der Graaf WT Groothuis DG Knell AJ Mycobacterium ulcerans infection in Ashanti region, Ghana Trans R Soc Trop Med Hyg 1989 83 410 413 2617591 10.1016/0035-9203(89)90521-x

[B12] Suykerbuyk P Wambacq J Phanzu DM Haruna H Nakazawa Y Ooms K Kamango K Stragier P Singa JN Ekwanzala F De HE De MP Kestens L Portaels F Persistence of Mycobacterium ulcerans disease (Buruli Ulcer) in the historical focus of Kasongo Territory, the Democratic Republic of Congo Am J Trop Med Hyg 2009 81 888 894 19861627 10.4269/ajtmh.2009.09-0049

[B13] Noeske J Kuaban C Rondini S Sorlin P Ciaffi L Mbuagbaw J Portaels F Pluschke G Buruli ulcer disease in Cameroon rediscovered Am J Trop Med Hyg 2004 70 520 526 15155984

[B14] Debacker M Aguiar J Steunou C Zinsou C Meyers WM Scott JT Dramaix M Portaels F Mycobacterium ulcerans disease: role of age and gender in incidence and morbidity Trop Med Int Health 2004 9 1297 1304 15598261 10.1111/j.1365-3156.2004.01339.x

[B15] Debacker M Portaels F Aguiar J Steunou C Zinsou C Meyers W Dramaix M Risk factors for Buruli ulcer, Benin Emerg Infect Dis 2006 12 1325 1331 17073079 10.3201/eid1209.050598 PMC3294727

[B16] Pouillot R Matias G Wondje CM Portaels F Valin N Ngos F Njikap A Marsollier L Fontanet A Eyangoh S Risk factors for buruli ulcer: a case control study in Cameroon PLoS Negl Trop Dis 2007 1 e101 18160977 10.1371/journal.pntd.0000101 PMC2154388

[B17] Raghunathan PL Whitney EA Asamoa K Stienstra Y Taylor TH Amofah GK Ofori-Adjei D Dobos K Guarner J Martin S Pathak S Klutse E Etuaful S van der Graaf WT van der Werf TS King CH Tappero JW Ashford DA Risk factors for Buruli ulcer disease (Mycobacterium ulcerans Infection): results from a case–control study in Ghana Clin Infect Dis 2005 40 1445 1453 15844067 10.1086/429623

[B18] Aiga H Amano T Cairncross S Adomako J Nanas OK Coleman S Assessing water-related risk factors for Buruli ulcer: a case–control study in Ghana Am J Trop Med Hyg 2004 71 387 392 15516631

[B19] Bayley AC Buruli ulcer in Ghana Br Med J 1971 2 401 402 5575986 10.1136/bmj.2.5758.401-c PMC1795769

[B20] Grietens KP Boock AU Peeters H Hausmann-Muela S Toomer E Ribera JM "It is me who endures but my family that suffers": social isolation as a consequence of the household cost burden of Buruli ulcer free of charge hospital treatment PLoS Negl Trop Dis 2008 2 e321 18923711 10.1371/journal.pntd.0000321 PMC2562517

[B21] Asiedu K Etuaful S Socioeconomic implications of Buruli ulcer in Ghana: a three-year review Am J Trop Med Hyg 1998 59 1015 1022 9886216 10.4269/ajtmh.1998.59.1015

[B22] Renzaho AM Woods PV Ackumey MM Harvey SK Kotin J Community-based study on knowledge, attitude and practice on the mode of transmission, prevention and treatment of the Buruli ulcer in Ga West District, Ghana Trop Med Int Health 2007 12 445 458 17313516 10.1111/j.1365-3156.2006.01795.x

[B23] Ackumey MM Kwakye-Maclean C Ampadu EO de Savigny D Weiss MG Health services for Buruli ulcer control: lessons from a field study in Ghana PLoS Negl Trop Dis 2011 5 e1187 21713021 10.1371/journal.pntd.0001187 PMC3119641

[B24] Stienstra Y van der Graaf WT Asamoa K van der Werf TS Beliefs and attitudes toward Buruli ulcer in Ghana Am J Trop Med Hyg 2002 67 207 213 12389949 10.4269/ajtmh.2002.67.207

[B25] Aujoulat I Johnson C Zinsou C Guedenon A Portaels F Psychosocial aspects of health seeking behaviours of patients with Buruli ulcer in southern Benin Trop Med Int Health 2003 8 750 759 12869098 10.1046/j.1365-3156.2003.01089.x

[B26] Ga-South Municipal Directorate Greater Accra - Weija Municipal Demographic Characteristics 2011 http://www.ghanadistricts.com/districts/?r=1&_=169&sa=6052

[B27] WHO, Global Buruli Ulcer Initiative Recognizing Buruli ulcer in your community 1998 World Health Organization, Geneva

[B28] Weiss MG Explanatory Model Interview Catalogue (EMIC): Framework for Comparative Study of Illness Transcult Psychiatry 1997 34 235 263

[B29] Agbenorku P Agbenorku M Amankwa A Tuuli L Saunderson P Factors enhancing the control of Buruli ulcer in the Bomfa communities, Ghana Trans R Soc Trop Med Hyg 2011 105 459 465 21652052 10.1016/j.trstmh.2011.05.003

[B30] Adamba C Owusu YA Burden of Buruli Ulcer: How Affected Households in a Ghanaian District Cope African Study Monographs 2011 32 1 23

[B31] Muela RJ Peeters GK Toomer E Hausmann-Muela S A word of caution against the stigma trend in neglected tropical disease research and control PLoS Negl Trop Dis 2009 3 e445 19859533 10.1371/journal.pntd.0000445 PMC2761539

[B32] Stienstra Y Dijkstra PU Guedenon A Johnson RC Ampadu EO Mensah T Klutse EY Etuaful S Deepak S van der Graaf WT van der Werf TS Development of a questionnaire assessing Buruli ulcer-induced functional limitation Am J Trop Med Hyg 2004 70 318 322 15031524

[B33] Government of Ghana and Ministry of Youth and Sports The National Youth Employment Programme (NYEP) 2011 http://www.ghana.gov.gh/index.php?option=com_content&view=article&id=5381:ministry-of-youth-and-sports&catid=74:ministries&Itemid=224

[B34] Walsh DS Portaels F Wayne M Buruli Ulcer (Mycobacterium ulcerans Infection): a Re-emerging Disease Clinical Microbiology Newsletter 2009 31 119 127

[B35] Gordillo V Del AJ Soriano V Gonzalez-Lahoz J Sociodemographic and psychological variables influencing adherence to antiretroviral therapy AIDS 1999 13 1763 1769 10509579 10.1097/00002030-199909100-00021

[B36] Macq J Torfoss T Getahun H Patient empowerment in tuberculosis control: reflecting on past documented experiences Trop Med Int Health 2007 12 873 885 17596255 10.1111/j.1365-3156.2007.01858.x

[B37] Johansson E Winkvist A Trust and transparency in human encounters in tuberculosis control: lessons learned from Vietnam Qual Health Res 2002 12 473 491 11939249 10.1177/104973202129120025

[B38] Yirga D Deribe K Woldemichael K Wondafrash M Kassahun W Factors associated with compliance with community directed treatment with ivermectin for onchocerciasis control in Southwestern Ethiopia Parasit Vectors 2010 3 48 20525182 10.1186/1756-3305-3-48 PMC2896929

[B39] Morisky DE Malotte CK Ebin V Davidson P Cabrera D Trout PT Coly A Behavioral interventions for the control of tuberculosis among adolescents Public Health Rep 2001 116 568 574 12196616 10.1016/S0033-3549(04)50089-4 PMC1497389

[B40] Worley S Didiza Z Nomatshila S Porter S Makwedini N Macharia D Hoos D Wellness programmes for persons living with HIV/AIDS: experiences from Eastern Cape province, South Africa Glob Public Health 2009 4 367 385 19343601 10.1080/17441690801994301

[B41] Ackumey MM Gyapong M Pappoe M Weiss MG Help-seeking for pre-ulcer and ulcer conditions of Mycobacterium ulcerans disease (Buruli ulcer) in Ghana. Am J Trop Med Hyg 2011 85 1106 1113 22144453 10.4269/ajtmh.2011.11-0429 PMC3225161

[B42] Vandelannoote K Durnez L Amissah D Gryseels S Dodoo A Yeboah S Addo P Eddyani M Leirs H Ablordey A Portaels F Application of real-time PCR in Ghana, a Buruli ulcer-endemic country, confirms the presence of Mycobacterium ulcerans in the environment FEMS Microbiol Lett 2010 304 191 194 20146745 10.1111/j.1574-6968.2010.01902.x

[B43] Mulder AA Boerma RP Barogui Y Zinsou C Johnson RC Gbovi J van der Werf TS Stienstra Y Healthcare seeking behaviour for Buruli ulcer in Benin: a model to capture therapy choice of patients and healthy community members Trans R Soc Trop Med Hyg 2008 102 912 920 18617207 10.1016/j.trstmh.2008.05.026

[B44] Etuaful S Carbonnelle B Grosset J Lucas S Horsfield C Phillips R Evans M Ofori-Adjei D Klustse E Owusu-Boateng J Amedofu GK Awuah P Ampadu E Amofah G Asiedu K Wansbrough-Jones M Efficacy of the combination rifampin-streptomycin in preventing growth of Mycobacterium ulcerans in early lesions of Buruli ulcer in humans Antimicrob Agents Chemother 2005 49 3182 3186 16048922 10.1128/AAC.49.8.3182-3186.2005 PMC1196249

[B45] Nienhuis WA Stienstra Y Thompson WA Awuah PC Abass KM Tuah W Awua-Boateng NY Ampadu EO Siegmund V Schouten JP Adjei O Bretzel G van der Werf TS Antimicrobial treatment for early, limited Mycobacterium ulcerans infection: a randomised controlled trial Lancet 2010 375 664 672 20137805 10.1016/S0140-6736(09)61962-0

[B46] Chauty A Ardant MF Adeye A Euverte H Guedenon A Johnson C Aubry J Nuermberger E Grosset J Promising clinical efficacy of streptomycin-rifampin combination for treatment of buruli ulcer (Mycobacterium ulcerans disease) Antimicrob Agents Chemother 2007 51 4029 4035 17526760 10.1128/AAC.00175-07 PMC2151409

[B47] Nyonator FK Awoonor-Williams JK Phillips JF Jones TC Miller RA The Ghana community-based health planning and services initiative for scaling up service delivery innovation Health Policy Plan 2005 20 25 34 15689427 10.1093/heapol/czi003

[B48] Brieger WR Health education to promote community involvement in the control of tropical diseases Acta Trop 1996 61 93 106 8740888 10.1016/0001-706x(95)00104-m

[B49] Cairncross S Braide EI Bugri SZ Community participation in the eradication of guinea worm disease Acta Trop 1996 61 121 136 8740890 10.1016/0001-706x(95)00106-o

[B50] Sagbakken M Frich JC Bjune G Barriers and enablers in the management of tuberculosis treatment in Addis Ababa, Ethiopia: a qualitative study BMC Public Health 2008 8 11 18186946 10.1186/1471-2458-8-11 PMC2257959

[B51] van der Werf TS Stienstra Y Johnson RC Phillips R Adjei O Fleischer B Wansbrough-Jones MH Johnson PD Portaels F van der Graaf WT Asiedu K Mycobacterium ulcerans disease Bull World Health Organ 2005 83 785 791 16283056 PMC2626418

[B52] World Health Organization Neglected tropical diseases, hidden successes, emerging opportunities 2009 World Health Organization, Geneva

[B53] Ga-West Municipality-Agricultural Sector http://www.ghanadistricts.com/districts/?r=1&_=1&sa=1140

[B54] Williamson HR Benbow ME Nguyen KD Beachboard DC Kimbirauskas RK McIntosh MD Quaye C Ampadu EO Boakye D Merritt RW Small PL Distribution of Mycobacterium ulcerans in buruli ulcer endemic and non-endemic aquatic sites in Ghana PLoS Negl Trop Dis 2008 2 e205 18365034 10.1371/journal.pntd.0000205 PMC2268743

[B55] Johnson PD Stinear TP Hayman JA Mycobacterium ulcerans–a mini-review J Med Microbiol 1999 48 511 513 10359298 10.1099/00222615-48-6-511

[B56] Johnson RC Makoutode M Sopoh GE Elsen P Gbovi J Pouteau LH Meyers WM Boko M Portaels F Buruli ulcer distribution in Benin Emerg Infect Dis 2005 11 500 501 15789490 10.3201/eid1103.040597 PMC3298242

[B57] Duker AA Portaels F Hale M Pathways of Mycobacterium ulcerans infection: a review Environ Int 2006 32 567 573 16492390 10.1016/j.envint.2006.01.002

[B58] The Uganda Buruli Group Epidemiology of Mycobacterium ulcerans infection (Buruli ulcer) at Kinyara, Uganda Trans R Soc Trop Med Hyg 1971 65 763 775 5157438 10.1016/0035-9203(71)90090-3

[B59] Asiedu K Portaels F Asiedu K, Scherpbier R, Raviglione MC Chapter One: Introduction Buruli ulcer:Mycobacterium ulcerans infection 2000 World Health Organisation, Geneva 5 7

[B60] Merritt RW Walker ED Small PL Wallace JR Johnson PD Benbow ME Boakye DA Ecology and transmission of Buruli ulcer disease: a systematic review PLoS Negl Trop Dis 2010 4 e911 21179505 10.1371/journal.pntd.0000911 PMC3001905

[B61] Fyfe JA Lavender CJ Handasyde KA Legione AR O'Brien CR Stinear TP Pidot SJ Seemann T Benbow ME Wallace JR McCowan C Johnson PD A major role for mammals in the ecology of Mycobacterium ulcerans PLoS Negl Trop Dis 2010 4 e791 20706592 10.1371/journal.pntd.0000791 PMC2919402

[B62] Portaels F Elsen P Guimaraes-Peres A Fonteyne PA Meyers WM Insects in the transmission of Mycobacterium ulcerans infection Lancet 1999 353 986 10459918 10.1016/S0140-6736(98)05177-0

